# Metabolic engineering of *Pichia pastoris* for production of isobutanol and isobutyl acetate

**DOI:** 10.1186/s13068-017-1003-x

**Published:** 2018-01-08

**Authors:** Wiparat Siripong, Philipp Wolf, Theodora Puspowangi Kusumoputri, Joe James Downes, Kanokarn Kocharin, Sutipa Tanapongpipat, Weerawat Runguphan

**Affiliations:** 1 National Center for Genetic Engineering and Biotechnology, 113 Thailand Science Park, Paholyothin Road, Klong 1, Klong Luang, Pathumthani 12120 Thailand; 2 Leipzig University, Brüderstraße 34, 04103 Leipzig, Germany; 3 Atma Jaya University, Jl. Jend. Sudirman No.51, RT.5/RW.4, Karet Semanggi, Setia Budi, Kota Jakarta Selatan, Daerah Khusus Ibukota Jakarta, 12930 Indonesia; 4 University of Kent, Canterbury, Kent CT2 7NZ UK

**Keywords:** Metabolic engineering, Isobutanol, Isobutyl acetate, Yeast

## Abstract

**Background:**

Interests in renewable fuels have exploded in recent years as the serious effects of global climate change become apparent. Microbial production of high-energy fuels by economically efficient bioprocesses has emerged as an attractive alternative to the traditional production of transportation fuels. Here, we engineered *Pichia pastoris*, an industrial workhorse in heterologous enzyme production, to produce the biofuel isobutanol from two renewable carbon sources, glucose and glycerol. Our strategy exploited the yeast’s amino acid biosynthetic pathway and diverted the amino acid intermediates to the 2-keto acid degradation pathway for higher alcohol production. To further demonstrate the versatility of our yeast platform, we incorporated a broad-substrate-range alcohol-*O*-acyltransferase to generate a variety of volatile esters, including isobutyl acetate ester and isopentyl acetate ester.

**Results:**

The engineered strain overexpressing the keto-acid degradation pathway was able to produce 284 mg/L of isobutanol when supplemented with 2-ketoisovalerate. To improve the production of isobutanol and eliminate the need to supplement the production media with the expensive 2-ketoisovalerate intermediate, we overexpressed a portion of the amino acid l-valine biosynthetic pathway in the engineered strain. While heterologous expression of the pathway genes from the yeast *Saccharomyces cerevisiae* did not lead to improvement in isobutanol production in the engineered *P. pastoris*, overexpression of the endogenous l-valine biosynthetic pathway genes led to a strain that is able to produce 0.89 g/L of isobutanol. Fine-tuning the expression of bottleneck enzymes by employing an episomal plasmid-based expression system further improved the production titer of isobutanol to 2.22 g/L, a 43-fold improvement from the levels observed in the original strain. Finally, heterologous expression of a broad-substrate-range alcohol-*O*-acyltransferase led to the production of isobutyl acetate ester and isopentyl acetate ester at 51 and 24 mg/L, respectively.

**Conclusions:**

In this study, we engineered high-level production of the biofuel isobutanol and the corresponding acetate ester by *P. pastoris* from readily available carbon sources. We envision that our work will provide an economic route to this important class of compounds and establish *P. pastoris* as a versatile production platform for fuels and chemicals.

**Electronic supplementary material:**

The online version of this article (10.1186/s13068-017-1003-x) contains supplementary material, which is available to authorized users.

## Background

Unsustainable demands and concerns over climate change have inspired interest in renewable fuels and chemicals [1–3]. In recent years, microbial production of high-energy fuels via environmentally sustainable and economically efficient bioprocesses has emerged as a viable alternative to the traditional production of transportation fuels [4]. Although microbial fermentation of ethanol has played an important role for the transition to bio-based fuels, ethanol is not an ideal gasoline replacement due to its poor physicochemical properties. Specifically, ethanol has low energy density (~ 70% of the energy content of gasoline), high hygroscopicity and high vapor pressure [3]. In contrast, branched-chain and higher alcohols, such as isobutanol and isoamyl alcohol, have superior energy density (at 90 and 110% of the energy content of gasoline, respectively) and are more compatible with the existing storage and distribution infrastructures. Moreover, branched-chain alcohols have higher octane numbers compared with their straight-chain counterparts and are therefore ideal gasoline substitutes for high performance petrol engines [5]. A closely related class of compounds to branched-chain and higher alcohols is volatile esters. Importantly, because volatile esters typically contain either a floral or fruity scent, they are highly sought-after by the fragrance and cosmetic industries, which together account for a large global market of $16.6 billion in 2012 (http://www.ialconsultants.com/). Moreover, these esters can also be applied to solvents, coatings and paints.

Despite significant efforts in using metabolic engineering to improve natural producers of these alcohols and esters in the past, there has been little success in reaching commercially relevant titers and productivity [6]. Indeed, commercial production of the vast majority of these alcohols in native organisms such as several *Clostridium* species is not economically feasible at present [5]. Other disadvantages of using *Clostridium* species as a production host include their slow growth, their intolerance to oxygen and their production of the byproducts butyrate, acetone and ethanol [7]. Therefore, development of a more efficient production platform in a non-native host for higher branched-chain alcohols and the corresponding acetate esters is urgently needed.

**Fig. 1:**
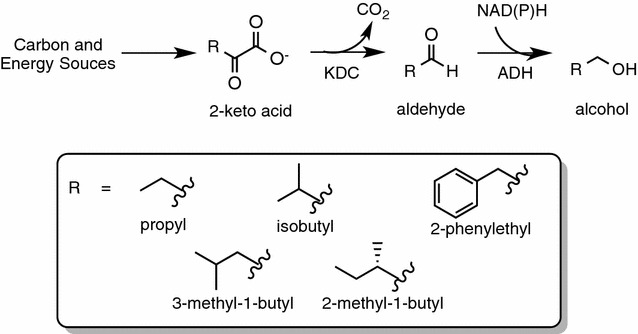
Construction of higher alcohol synthesis pathways in heterologous hosts such as *E. coli* [8], *S. cerevisiae* [12, 14] and *P. pastoris* (this work). In vivo higher branched-chain alcohol production is derived from 2-keto acid. *KDC* keto acid decarboxylase, *ADH* alcohol dehydrogenase

Atsumi and co-workers developed a non-fermentative pathway for producing branched-chain higher alcohols in *Escherichia coli* [8] (Table 1). This synthetic pathway involves “hijacking” the native amino acid metabolism of the bacterial host (Fig. 1). Specifically, 2-keto acids, which are intermediates in amino acid biosynthesis, are converted to aldehydes by introducing a heterologous keto acid decarboxylase (KDC) from the bacterium *Lactococcus lactis*. The resulting aldehydes are then reduced by an alcohol dehydrogenase (ADH) from *Saccharomyces cerevisiae* to the final alcohol products, including isobutanol, 1-butanol, 2-methyl-1-butanol, 3-methyl-1-butanol (isoamyl alcohol), and 2-phenyl-ethanol. Further analysis indicated that the endogenous alcohol dehydrogenase YqhD in *E. coli* was superior to the yeast enzyme ADH2 at converting isobutanal to isobutanol and was employed in subsequent work to enhance the yield of isobutanol [9]. Remarkably, overexpression of genes responsible for the biosynthesis of the l-valine precursor 2-ketoisovalerate (C5), in conjunction with substitutions of genes from other organisms and deletion of several genes from competing pathways, resulted in the production of up to 22 g/L isobutanol in shake flasks, 86% of the theoretical maximum [8]. The cyanobacterium *Synechococcus elongatus* PCC7942 has also been explored as a production host for higher alcohol production [10]. By introducing the synthetic non-fermentative pathway genes from several species into *S. elongatus* PCC7942, Atsumi and co-workers were able to produce isobutanol directly from carbon dioxide, though with relatively modest titer of 0.450 g/L. In addition to the prokaryotic systems, the Baker’s yeast *Saccharomyces cerevisiae* has also been actively explored as a potential production platform for higher alcohols [11–16]. By upregulating the endogenous valine metabolism in *S. cerevisiae*, Chen and co-workers were able to attain an isobutanol yield of 3.86 mg/g glucose [13]. Higher yields were achieved when the upstream pathway (valine metabolism, up to 2-keto acid) and downstream pathway (2-keto acid degradation) were co-localized in the same organelles (either mitochondria or cytosol), with 16.00 mg/g glucose being the highest yield reported so far in the literature [11, 14].

**Table 1:** Overview of different microbial strains including *Pichia pastoris* (our work) engineered to produce isobutanol and isobutyl acetate

Strain name/host strain	Overexpressed genes	Carbon source (g/L)	Specific growth rate (h^−1^)	Product	Average titer (g/L)	Yield (mg/g carbon source)	Productivity (mg/L h)/maximum productivity (mg/L h)	References
*Escherichia coli*								
JCL260	*LlkivD, ScADH2, BsalsS, ilvCD*	Glucose (36)	Not reported	Isobutanol	22	350 (between 40 and 112 h)	196.4	Atsumi et al. [8]
JCL260 Δ*poxB*	*LlkivD, ScADH2, BsalsS, ilvCD*	Glucose (fed batch fermentation)	~ 0.6	Isobutanol	50.8 ± 1.1^a^	290^a^	705.6^a^	Baez et al. [36]
*Synechococcus elongatus*								
PCC7942	*LlkivD, EcYqhD, BsalsS, EcilvCD Rubisco*	Carbon dioxide	Not reported	Isobutanol	0.45	Not reported	3.12	Atsumi et al. [10]
*Saccharomyces cerevisiae*								
CEN.PK2-1C *Δilv2*	*ScIlv2_cytosolic, ScIlv3_cytosolic, ScIlv5_cytosolic, ScARO10, ScADH2*	Glucose (40)	Not reported	Isobutanol	0.630	14.18	6.56	Brat et al. [15]
BY4741 × Y3929 (diploid)	*ScIlv2, ScIlv3, ScIlv5, ScARO10_mitochondrial, adhA_mitochondrial*	Glucose (100)	Not reported	Isobutanol	0.635 ± 0.023	6.7 ± 0.2	20.5 ± 1.2	Avalos et al. [14]
YPH499 *Δlpd1*	*ScIlv2, ScIlv2_cytosolic, ScIlv3_cytosolic, ScIlv5_cytosolic, LlkivD, ScADH6, ScMAE1*	Glucose (100) (Semi-anaerobic)	Not reported	Isobutanol	1.620	16.00	67.5	Matsuda et al. [11]
*Pichia pastoris*								
KM71	None	Glucose (100)	0.120 ± 0.002	Isobutanol	0.037 ± 0.005	0.37	0.52/1.22	This study
PP100	*LlkivD, ScADH7*	Glucose (100)	0.122 ± 0.001	Isobutanol	0.049 ± 0.006	0.49	0.68/1.68	This study
PP200	*LlkivD, ScADH7, PpIlv5, PpIlv3*	Glucose (100)	0.123 ± 0.001	Isobutanol	0.048 ± 0.001	0.48	0.66/1.98	This study
PP300	*LlkivD, ScADH7, PpIlv5, PpIlv3, PpIlv6* (codon optimized), *PpIlv2* (codon optimized)	Glucose (100)	0.107 ± 0.002	Isobutanol	0.885 ± 0.011	8.85	12.29/31.80	This study
PP300	LlkivD, ScADH7, PpIlv5, PpIlv3, PpIlv6 (codon optimized), PpIlv2 (codon optimized)	Glucose (20)	0.107 ± 0.001	Isobutanol	0.200 ± 0.001	2.00	2.78/9.60	This study
PP310	*LlkivD*, *ScADH7, PpIlv5, PpIlv3, PpIlv6* (codon optimized)*, PpIlv2* (codon optimized) and further overexpression of *PpIlv6* and *PpIlv2* by integration of another copy of the gene cassette	Glucose (100)	0.092 ± 0.002	Isobutanol	1.699 ± 0.073	16.99	23.60/60.41	This study
PP302	*LlkivD*, *ScADH7, PpIlv5, PpIlv3, PpIlv6* (codon optimized) and *PpIlv2* (codon optimized) and further episomal-plasmid based expression of *LlkivD* and *ScADH7*	Glucose (100)	0.096 ± 0.001	Isobutanol	1.716 ± 0.054	17.16	23.83/60.92	This study
PP303	*LlkivD*, *ScADH7, PpIlv5, PpIlv3, PpIlv6* (codon optimized) and *PpIlv2* (codon optimized) and further episomal-plasmid based expression of *PpIlv5* and *PpIlv3*	Glucose (100)	0.108 ± 0.004	Isobutanol	1.745 ± 0.090	17.45	24.23/67.97	This study
PP304	*LlkivD*, *ScADH7, PpIlv5, PpIlv3, PpIlv6* (codon optimized) and *PpIlv2* (codon optimized) and further episomal-plasmid based expression of *PpIlv6* and *PpIlv2*	Glucose (100)	0.109 ± 0.002	Isobutanol	2.221 ± 0.048	22.21	30.84/60.88	This study
PP400	LlkivD, ScADH7, PpIlv5, PpIlv3, PpIlv6 (codon optimized), PpIlv2 (codon optimized) and ScATF1	Glucose (100)	0.099 ± 0.001	Isobutyl acetate	0.006 ± 0.001	0.06	0.06	This study
PP401	*LlkivD*, *ScADH7, PpIlv5, PpIlv3, PpIlv6* (codon optimized)*, PpIlv2* (codon optimized) and episomal-plasmid based expression of *ScATF1*	Glucose (100)	0.091 ± 0.001	Isobutyl acetate	0.051 ± 0.007	0.51	0.53	This study

The reported average titers do not take into account different fermentation times and conditions and are therefore not directly comparable

^a^Fermentation was performed in a 1-L bioreactor equipped with a gas stripping system for in situ product removal

Despite these considerable successes, *E. coli* and *S. cerevisiae* suffer from several disadvantages that make them unfavorable as production hosts for isobutanol and other higher alcohols. *E. coli* is susceptible to viral or bacteriophage infections [17]. On the other hand, the Crabtree-positive *S. cerevisiae* suffers from low production yield due to the yeast’s inherent preference for ethanol production under high glucose conditions [18, 19]. While several strategies exist to minimize ethanol formation by engineered *S. cerevisiae*, most efforts only led to moderate increase in isobutanol yields [12]. Given the shortcomings associated with higher alcohols production in *E. coli* and *S. cerevisiae*, we seek to establish *Pichia pastoris*, a Crabtree-negative methylotrophic yeast used industrially to produce recombinant proteins, as a production host for higher alcohols and the corresponding acetate esters.

For over a decade, *P. pastoris* has been extensively engineered to produce many industrially relevant enzymes such as xylanase, phytase, human serum albumin, hepatitis B surface antigen and glycoproteins with human-type N-glycosylation, many of which are already on the market [20, 21]. In particular, high-level heterologous expressions of various cellulase enzymes with comparable stability and activity to the native host have been achieved in *P. pastoris* [22–24]. In a recent example, Varnai and coworkers were able to obtain 3–5 g/L of secreted fungal endoglucanases using a recombinant *P. pastoris* system [22]. We contend that the demonstrated robustness of *P. pastoris* in heterologous expression of cellulolytic and hemicellulolytic enzymes should make the organism an attractive host for consolidated bioprocessing of biofuels and other chemicals from biomass.

Despite the proven versatility of *P. pastoris* in industrial settings, biotechnological applications of the yeast have been mostly limited to recombinant enzyme expression. Indeed, examples of harnessing the yeast to produce industrially relevant biofuels and chemicals are scarce when compared to other industrial hosts such as *S. cerevisiae* and *E. coli*. In a recent example, *P. pastoris* was engineered to produce nootkatone, a sesquiterpene in great commercial demand for its aroma, by overexpressing the sesquiterpenoid pathway [25]. However, despite this success, the best-engineered *P. pastoris* strain only produces ~ 200 mg/L of nootkatone in bioreactor cultivations. This titer is still an order of magnitude below a commercially relevant level.

In this work, we engineered *P. pastoris* to produce the advanced biofuel isobutanol and its corresponding ester, isobutyl acetate ester. We exploited *P. pastoris*’ endogenous amino acid biosynthetic pathway and channeled the amino acid intermediate, 2-ketoisovalerate, to the 2-keto acid degradation pathway for isobutanol production. Overexpression of the endogenous l-valine biosynthetic pathway genes led to a strain that is able to produce approximately 0.89 g/L of isobutanol directly from glucose. Further improvement of the production titer to more than 2.2 g/L was achieved by employing an episomal-plasmid based expression system to fine-tune the expression of all pathway enzymes. Finally, to demonstrate the versatility of our yeast platform, we further incorporated a broad-substrate-range alcohol *O*-acyltransferase to generate isobutyl acetate ester. We envision that our yeast platform will pave the way for an economic route to biofuels and chemicals.

## Methods

### Yeast strain, media and transformation

The yeast strains used in this study were constructed from *P. pastoris* KM71 (Invitrogen) (Table 1; Additional file 1: Table S1). The plasmids used in this study were generated from the pGAPZ_A and pPIC3.5K vectors (Invitrogen). Detailed description of plasmid construction is provided in Additional file 1. Yeast transformation was performed as previously described using an electroporator [26]. The following parameters were used for the electroporation: 1.5 kV, 25 μF, 200 Ω. Colony PCR and DNA sequencing were used to verify strain construction. Yeast and bacterial strains were stored in 25% glycerol at − 80 °C. *E. coli* was grown in Luria–Bertani medium supplemented with ampicillin (at 100 μg/L), hygromycin (at 100 μg/L), kanamycin (at 50 μg/L), or zeocin (at 100 μg/L) when required. *P. pastoris* was grown in YPD medium (10 g/L yeast extract, 20 g/L Bacto Peptone and 20 g/L glucose) supplemented with hygromycin (at 200 μg/L), G418 (at 100 μg/L), or zeocin (at 100 μg/L) when required. Selection of *P. pastoris* transformants with HIS4 was done on a yeast minimal medium [MGY (pH 6.0) containing: 13.6 g/L yeast nitrogen base without amino acids, 20 g/L glycerol, 0.1 M phosphate buffer, 0.4 mg/L d-biotin, 133.3 mg/L thiamine-hydrochloride].

### Screening of KDC and ADH candidate enzymes

Characterization of engineered *P. pastoris* strains overexpressing various KDC and ADH enzymes was carried out in a minimal medium [MGYH (pH 6.0) containing: 13.6 g/L yeast nitrogen base without amino acids, 20 g/L glycerol, 0.1 M phosphate buffer, 0.4 mg/L d-biotin, 133.3 mg/L thiaminchloride–hydrochloride, and 20 mg/L l-histidine]. Briefly, engineered strains were pre-cultured in 5-mL aliquots in MGYH medium overnight and used to inoculate 10 mL MGYH medium (either with or without 4 g/L 2-ketoisovalerate) in 50 mL Corning tubes to achieve an initial optical density of 0.05 at 600 nm (OD_600_). The cultures were grown at 30 °C and 250 rpm in an orbital shaking incubator. Samples were taken at 24 and 48 h to determine OD_600_, extracellular metabolites and production of higher alcohols.

The amount of isobutanol and other extracellular metabolites were determined using high-performance liquid chromatography (HPLC). Briefly, 1 mL of culture was centrifuged at 18,000*g* for 5 min and the supernatant was filtered through 0.45 μm nylon syringe filter (Filtrex). The purified sample was then applied to an Agilent 1100 series HPLC equipped with an Aminex HPX-87H ion exchange column (Biorad). The LC program was performed using 5 mM H_2_SO_4_ as the solvent at a flow rate of 0.72 mL/min for 42 min. The column was maintained at 55 °C. All metabolites were detected with Agilent 1200 series DAD and RID detectors.

### Quantification of isobutanol production in engineered strains

Characterization of engineered *P. pastoris* strains was carried out in a minimal medium as described in “Screening of KDC and ADH candidate enzymes” section. l-Histidine was omitted from the medium when HIS4 was used as the selection marker; this medium is designated MGY. In the study comparing the different carbon sources, glycerol was replaced with glucose, resulting in a modified MGYH with glucose as a sole carbon source (designated MGYH-glu). In the study comparing different concentrations of glucose, the media were prepared with either 2 or 10% glucose and were designated MGYH-glu (2% glucose) and MGYH-glu (10% glucose), respectively.

Shake flask fermentation was also carried out in selected strains (KM71, PP100, PP200, PP300, PP310, PP302, PP303 and PP304). For this, engineered strains were pre-cultured in 5-mL aliquots in MGYH medium overnight and used to inoculate 50 mL MGYH-glu medium (at either 2 or 10% glucose as indicated) in 250 mL Erlenmeyer flasks closed with cotton wool plugs to achieve an initial optical density of 0.05 at 600 nm (OD_600_). The cultures were grown at 30 °C and 250 rpm in an orbital shaking incubator. Samples were taken to determine OD_600_, biomass, extracellular metabolites and production of higher alcohols. The amount of isobutanol and other extracellular metabolites were determined using HPLC as described above.

### Quantification of isobutyl acetate production in engineered strains

Quantification of isobutyl acetate ester in engineered strains was performed as previously described with some modifications [27]. Engineered strains were pre-cultured in 5-mL aliquots in MGYH overnight and used to inoculate 10 mL modified MGY-glu medium in 50 mL Corning tubes as described in “Screening of KDC and ADH candidate enzymes” section. The yeast cultures were overlaid with 10 mL hexadecane (Sigma) to reduce evaporation of the acetate esters. The cultures were grown at 30 °C and 250 rpm in an orbital shaking incubator. Samples were taken to determine biomass, extracellular metabolites and production of higher alcohols and acetate esters. The amount of acetate esters dissolved in the hexadecane layer was determined using gas chromatography–mass spectrometry (GC–MS). The GC program was as follows: an initial temperature of 40 °C was maintained for 4 min, followed by ramping to 300 °C at a rate of 45 °C per min, where the temperature was held for 3 min. The injector temperature was held at 250 °C. The injection volume was 5 μL, injected at a 10:1 split ratio. Hydrogen was used as the carrier gas. The MS is a GCMS-QP2010S (Shimadzu). The ion source temperature was 200 °C, and the interface temperature was 250 °C. The solvent cut time was 2 min. The start *m*/*z* was 40, and the end *m*/*z* was 500. Mass spectra and retention times from samples were compared with authentic standards (Sigma).

### RNA isolation and transcript quantification

Strains were pre-cultured in 5-mL aliquots in MGYH medium overnight and used to inoculate 10 mL modified MGYH-glu medium as described in “Screening of KDC and ADH candidate enzymes” section. After 24 and 48 h, a 5-mL aliquot of each culture was collected and centrifuged for 5 min at 3000*g*. The pellets were washed with 5 mL of distilled water. Total RNA was extracted using the QIAgen RNeasy Kit under the manufacturer’s protocol. Contaminating genomic DNA was removed from the RNA samples by DNaseI (NEB) digestion using the manufacturer’s protocol. The RNA quantity was analyzed using a NanoDrop ND-1000 spectrophotometer (NanoDrop Technologies), and samples were stored at − 80 °C until RT-PCR analysis. cDNA was obtained using RevertAid Reverse Transcriptase (Thermo Fisher Scientific) using the manufacturer’s protocol. Relative expression levels of *PpIlv6_CodOpt, PpIlv2_CodOpt, PpIlv5, PpIlv3, LlkivD_CodOpt, ScADH7* and *ScATF1* were quantified using iQ SYBR Green Kit (Biorad) on CFX96 Touch Real-time PCR Detection System (Biorad). Real-time PCR was performed in triplicates, and *PpACT1*, a gene that encodes actin, was used to normalize the amount of the total mRNA in all samples.

### Total DNA isolation and gene copy quantification

Strains were pre-cultured in 5-mL aliquots in MGY medium overnight in 50 mL Corning tubes as described in “Screening of KDC and ADH candidate enzymes” section. Total DNA was isolated using Wizard Genomic DNA purification kit (Promega) using the manufacturer’s protocol. The DNA quantity was assessed using a NanoDrop ND-1000 spectrophotometer (NanoDrop Technologies), and samples were stored at − 20 °C until real-time PCR analysis. Gene copy numbers of *PpIlv6_CodOpt, PpIlv2_CodOpt, PpIlv5, PpIlv3, LlkivD_CodOpt, ScADH7* and *ScATF1* were quantified using iQ SYBR Green Kit (Biorad) on CFX96 Touch Real-time PCR Detection System (Biorad) as previously reported with some modifications (Marx 2009). Real-time PCR was performed in triplicate, and normalization of the data was achieved using actin as a reference (i.e. *Pp*ACT1 gene copy number = 1). Primers used for RT-PCR are listed in Additional file 1.

## Results and discussion

### Overexpression of the 2-keto acid degradation pathway genes to enhance alcohol production from 2-keto acids

To metabolically engineer *P. pastoris* to produce higher alcohols, we employed a strategy that had been used successfully in *E. coli* and *S. cerevisiae* [8, 12–14]. Specifically, we exploited *P. pastoris’* endogenous amino acid biosynthetic pathway and channeled the amino acid intermediates to the 2-keto acid degradation pathway for higher alcohol production (Fig. 1). In the 2-keto acid degradation pathway (also known as the Ehrlich pathway), 2-keto acids are converted into higher alcohols in two enzymatic steps: decarboxylation of 2-keto acids to aldehydes by 2-keto acid decarboxylase (KDC), and subsequent reduction of aldehydes to alcohols by alcohol dehydrogenase (ADH).

We first searched for suitable KDC and ADH enzymes to enhance the endogenous activity of the Ehrlich pathway and to improve the higher alcohol production. Specifically, we screened three different candidates of KDC that have demonstrated high activity in either *E. coli* or *S. cerevisiae*. These are: KIVD from *Lactococcus lactis*, and ARO10 and THI3 from *S. cerevisiae* [8]. For ADH, we screened two enzymes—both from *S. cerevisiae*—that have different substrate specificities and catalytic properties. These are: ADH6 and ADH7 [12]. To assess the in vivo activities of these KDC and ADH candidates in *P. pastoris,* we supplemented the culture medium with 2-ketoisovalerate and quantified the production of isobutanol.

**Fig. 2:**
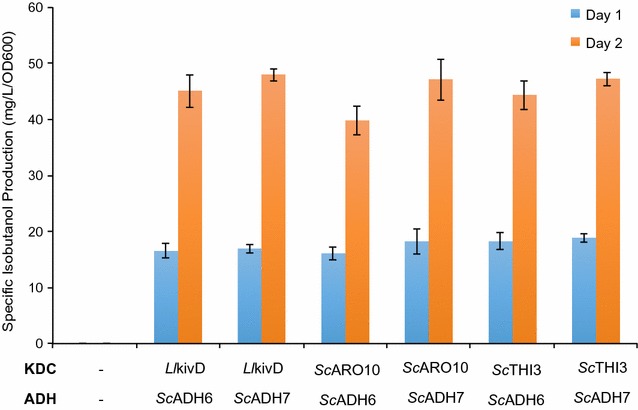
Screening of different keto acid decarboxylase and alcohol dehydrogenase variants. Engineered strains were pre-cultured in 5-mL aliquots in MGYH minimal medium overnight and used to inoculate 5 mL fresh MGYH [with glycerol as the main carbon source and supplemented with 4 g/L 2-ketoisovalerate (KIV)] to achieve an initial optical density of 0.05 at 600 nm (OD600). The cultures were grown at 30 °C and 250 rpm in an orbital shaking incubator. Samples were taken at 24 and 48 h time points and the supernatants were analyzed on HPLC to quantify the isobutanol content. Values are the mean of three biological replicates ± standard deviation (*n* = 3)

Gratifyingly, we observed isobutanol production when both KDC and ADH enzymes were expressed in *P. pastoris* and when the culture medium was supplemented with 2-ketoisovalerate (KIV, 4 g/L) (Fig. 2). The highest specific isobutanol titer—at 48 ± 1 mg/L/OD_600_—was observed when *L. lactis* KIVD and *S. cerevisiae* ADH7 were overexpressed. The specific titer decreased to approximately 40 ± 2 mg/L/OD_600_ when *S. cerevisiae* ARO10 and ADH6 were overexpressed. We were not able to detect any isobutanol in the control strain where none of the KDC and ADH enzymes was overexpressed, nor when KIV was absent from the medium (Fig. 2).

### Overexpression of l-valine biosynthetic pathway genes to further increase the pool of 2-ketoisovalerate and subsequently the production titer of isobutanol

**Fig. 3:**
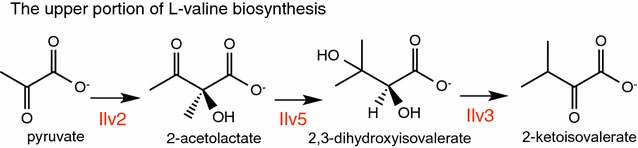
The upper portion of l-valine biosynthesis from pyruvate to 2-ketoisovalerate. Ilv2, acetolactate synthase; Ilv5, acetohydroxyacid reductoisomerase; and Ilv3, dihydroxyacid dehydratase. In *S. cerevisiae,* acetolactate synthase is encoded by one gene, *ScIlv2*, while in *P. pastoris*, the enzyme is encoded by two genes, *PpIlv2* and *PpIlv6* (small or regulatory subunit of acetolactate synthase)

Having determined *L. lactis* KIVD and *S. cerevisiae* ADH7 to be the most promising KDC and ADH enzyme candidates, respectively, we next turned our attention to increasing the production of the key intermediate KIV. Overproducing KIV not only obviates the need to supplement the media with expensive 2-keto acid, but has also been shown to markedly decrease the production of the other alcohols [8]. We increased the pool of KIV by upregulating a portion of l-valine biosynthesis (Fig. 3). In this pathway, acetolactate synthase condenses two molecules of pyruvate to 2-acetolactate, which is then reduced to 2,3-dihydroxyisovalerate by acetohydroxyacid reductoisomerase. Finally, 2,3-dihydroxyisovalerate is converted to KIV by dihydroxyacid dehydratase. In *P. pastoris*, acetolactate synthase, acetohydroxyacid reductoisomerase and dihydroxyacid dehydratase are encoded by four different genes, *PpIlv2*, *PpIlv6* (small subunit of acetolactate synthase), *PpIlv5* and *PpIlv3*, whereas in *S. cerevisiae*, the enzymes are encoded by just three genes, *ScIlv2*, *ScIlv5* and *ScIlv3*. Given the smaller number of genes required for overexpression in the case of *S. cerevisiae* vs. *P. pastoris* and the demonstrated activity of the enzymes in previous reports, we first attempted to overexpress the *S. cerevisiae* variants in the selected engineered *P. pastoris* strain with the enhanced 2-keto acid degradation pathway.

**Fig. 4:**
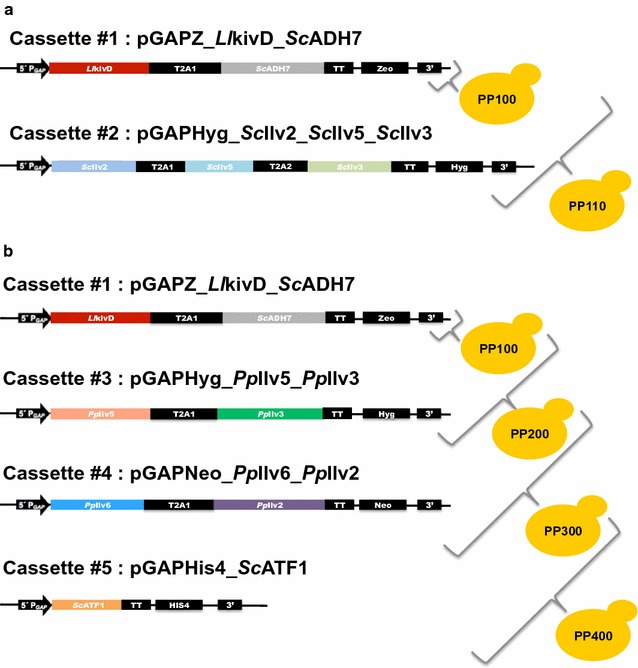
Construction of isobutanol and isobutyl acetate ester production strains. The individual genes in the keto-acid degradation and l-valine biosynthetic pathways together were linked together with the other members of the same pathway to create separate modules. For example, in the strain PP110 (**a**), *LlkivD* and *ScADH7* were linked together by self-cleaving 2A peptide sequence and placed behind the GAP promoter in the integrative expression plasmid, pGAP-Z; *Ilv2*, *Ilv5* and *Ilv3* (all from *S. cerevisiae*) were also linked together by self-cleaving 2A peptide sequence and placed behind the GAP promoter in the integrative expression plasmid, pGAP-Hyg. The individual constructs were sequentially integrated into the yeast genome to create PP110. Similarly, in the isobutanol producer strain PP300 (**b**), *LlkivD* and *ScADH7* were linked together by self-cleaving 2A peptide sequence and placed behind the GAP promoter in the integrative expression plasmid, pGAP-Z; *PpIlv6* and *PpIlv2* were linked together by self-cleaving 2A peptide sequence and placed behind the GAP promoter in the integrative expression plasmid, pGAP-Neo; *PpIlv5* and *PpIlv3*, were also linked together by self-cleaving 2A peptide sequence and placed behind the GAP promoter in the integrative expression plasmid, pGAP-Hyg. The individual constructs were sequentially integrated into the yeast genome to create PP300

Given the comparatively limited availability of genetic tools for heterologous expression of multiple genes in *P. pastoris*, we decided to link the individual genes in the keto-acid degradation and l-valine biosynthetic pathways together with the other members of the same pathway to create two separate modules (Fig. 4a). We chose to link the individual genes with the self-cleaving 2A peptide sequence from the foot-and-mouth disease virus (FMDV) to ensure stoichiometric production of all the proteins whose genes are linked together by the 2A peptide sequence [28]. A useful strategy expressing more than one protein, the 2A peptides allow multiple proteins to be encoded as polyproteins, which dissociate into individual proteins upon translation. Fermentation studies of the PP101 strain containing the three l-valine biosynthetic pathway genes from *S. cerevisiae* and the two 2-keto acid degradation pathway genes were performed in culture medium either with or without supplementation of KIV. Unfortunately, isobutanol production was observed only when KIV was supplemented to the culture media (Additional file 1: Figure S1). Negligible amounts of isobutanol were observed when the intermediate was absent from the media. Our results suggested that the l-valine biosynthetic pathway genes from *S. cerevisiae* could not be functionally expressed in *P. pastoris.*

**Fig. 5:**
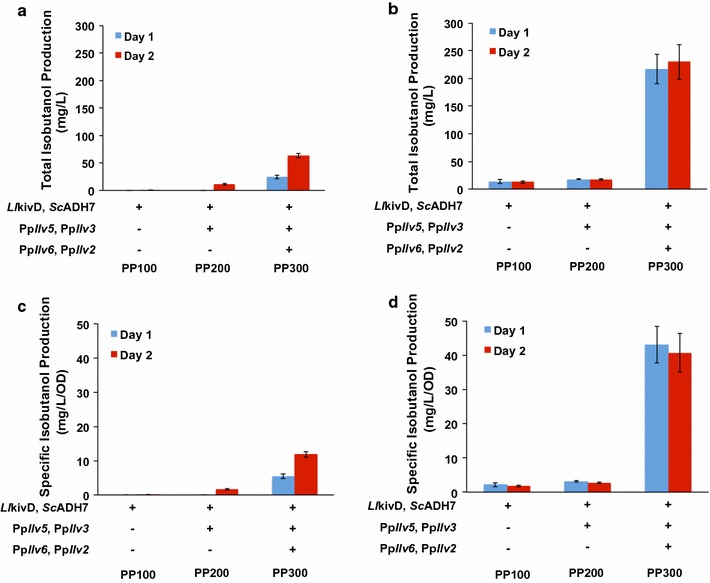
Effects of different carbon sources on isobutanol production in engineered *P. pastoris*. Total isobutanol production in minimum yeast media with either 2% glycerol (**a**) or 2% glucose (**b**) as the sole carbon source. Specific isobutanol production in minimum yeast media with either 2% glycerol (**c**) or 2% glucose (**d**) as the sole carbon source. Engineered strains (PP100, PP200, PP300) were pre-cultured in 5-mL aliquots in MGYH (2% glycerol) minimal medium overnight and used to inoculate either 5 mL fresh MGYH (2% glycerol, **a**, **c**) or 5 mL fresh MGYH_glu (2% glucose, **b**, **d**) to achieve an initial optical density of 0.05 at 600 nm (OD600). The cultures were grown at 30 °C and 250 rpm in an orbital shaking incubator. Samples were taken at 24 and 48 h time points and the supernatants were analyzed on HPLC to quantify the isobutanol content. Values are the mean of three biological replicates ± standard deviation (*n* = 3)

To circumvent this problem, we overexpressed *P. pastoris*’ endogenous l-valine biosynthetic pathway genes in the selected engineered *P. pastoris* strain with the enhanced 2-keto acid degradation pathway (Fig. 4b). The two genes, *PpIlv6* and *Pp*Ilv2 (each encodes a different subunit of the acetolactate synthase), were linked together by self-cleaving 2A peptide sequence and placed behind the *GAP* promoter in the integrative expression plasmid, pGAP-Neo. The remaining two genes, *Pp*Ilv5 and *Pp*Ilv3, were also linked together by self-cleaving 2A peptide sequence and placed behind the *GAP* promoter in the integrative expression plasmid, pGAP-Hyg. We observed isobutanol production in the engineered strain overexpressing the endogenous l-valine biosynthetic pathway genes. Specifically, PP300, the strain overexpressing all six genes (*LlkivD, ScADH7, PpIlv6, PpIlv2, PpIlv5* and *PpIlv3*) produced approximately 11.8 ± 0.8 mg/L/OD_600_ after 2 days in minimal yeast media with 2% glycerol, a 100-fold improvement over the levels observed in the strains overexpressing only the two keto acid degradation pathway genes, *Ll*kivD and *Sc*ADH7 (PP100; 0.12 ± 0.04 mg/L/OD_600_) (Fig. 5a, c). Low levels of isobutanol (1.6 ± 0.2 mg/L/OD_600_) were also observed in the strain PP200, which overexpresses both keto acid degradation pathway genes but only two of the l-valine biosynthetic pathway genes, *Pp*Ilv5 and *Pp*Ilv3. Our results suggested that expression of all four genes from the upper portion of l-valine pathway is required to observe high levels of isobutanol production.

**Fig. 6:**
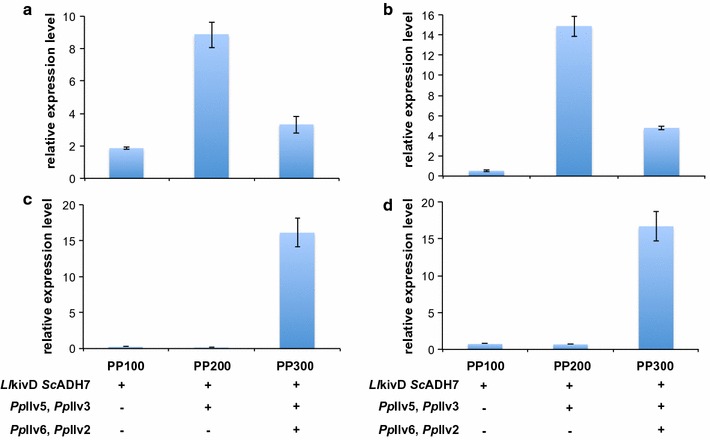
RT-PCR analysis of isobutanol biosynthetic pathway genes including *Pp*Ilv5 (**a**), *Pp*Ilv3 (**b**) *Pp*Ilv6 (**c**), *Pp*Ilv2 (**d**) in engineered yeast. The engineered strains PP100, PP200, and PP300 were pre-cultured in 5-mL aliquots in MGYH (2% glycerol) minimal medium overnight and used to inoculate either 10 mL fresh MGYH (2% glycerol) to achieve an initial optical density of 0.05 at 600 nm (OD600). The cultures were grown at 30 °C and 250 rpm in an orbital shaking incubator. Samples were taken at 48 h time points for real time RT-PCR analysis

In order to verify that the increase in isobutanol production correlated with increased expression levels of the l-valine pathway genes and the keto-acid degradation pathway genes, we performed RT-PCR in the three engineered strains, PP100, PP200 and PP300 (Fig. 6). The expression levels of all four genes (*Pp*Ilv5, *Pp*Ilv3, *Pp*Ilv6 and *Pp*Ilv2) were higher (3- to 16-folds) in PP300 compared to the levels observed in PP100. Interestingly, the expression levels of *Pp*Ilv5 and *Pp*Ilv3 were lower in PP300 compared to PP200 (Fig. 6a, b), indicating that usage of the same promoter (i.e. P_GAP_ in our case) to drive the expression of multiple genes led to lower expression levels of the individual genes.

### Analysis of the effects of carbon sources on isobutanol production

**Fig. 7:**
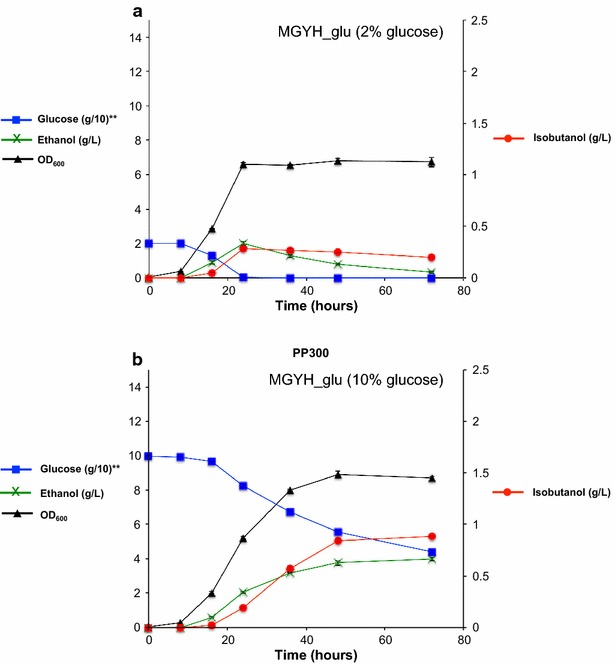
Effects of glucose concentration in the culture media on total isobutanol production in engineered *P. pastoris*. The engineered strain PP300 was pre-cultured in 5-mL aliquots in MGYH (2% glycerol) minimal medium overnight and used to inoculate either 50 mL fresh MGYH_glu (2% glucose) (**a**) or 50 mL fresh MGYH_glu (10% glucose) (**b**) in 250 mL Erlenmeyer flasks to achieve an initial optical density of 0.05 at 600 nm (OD600). The cultures were grown at 30 °C and 250 rpm in an orbital shaking incubator. Samples were taken at several time points and the supernatants were analyzed on HPLC to quantify the levels of isobutanol, glucose and other metabolites. Values are the mean of three biological replicates ± standard deviation (*n* = 3)

We next studied the effects of the concentration and type of carbon source on isobutanol production in our engineered yeast strains. When we switched the carbon source from 2% glycerol to 2% glucose (i.e. from MGYH to MGHY_glu), we observed an approximately fourfold improvement (from 11.8 ± 0.8 to 43.0 ± 5.3 mg/L/OD_600_) in isobutanol production (Fig. 5b, d). The elevated production level corresponds to the upregulation of all isobutanol biosynthetic pathway genes in the medium with glucose as the sole carbon source (Additional file 1: Figure S2). Interestingly, we observed that isobutanol production leveled off after 1 day in this medium (Fig. 5b, d), potentially due to glucose limitation and limited oxygen availability. To increase the oxygen availability, we performed our fermentation studies in 250-mL Erlenmeyer flasks instead of 50-mL conical tubes. When we increased the glucose concentration in the MGYH_glu medium from 2 to 10% as well as extending the shake flask fermentation time to 3 days, we observed a 3.1-fold improvement (from 282 ± 7 to 885 ± 12 mg/L) in total isobutanol production (Fig. 7). The elevated production level corresponds to the upregulation of all isobutanol biosynthetic pathway genes in the medium with the increased glucose concentration (Additional file 1: Figure S3). Accumulatively, our results indicated that the engineered *P. pastoris* strains are able to produce isobutanol in minimal yeast medium containing glucose as the sole carbon source.

### Improvement of isobutanol production by fine-tuning the expression of isobutanol biosynthetic pathway genes

**Fig. 8:**
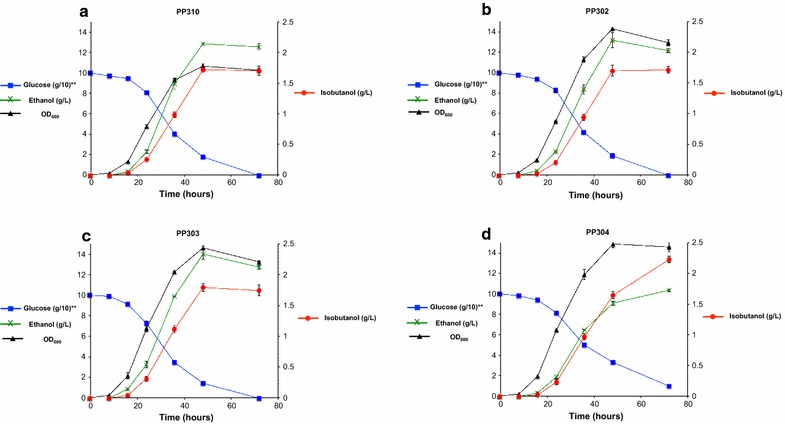
Strain improvement by varying isobutanol pathway gene copy number. **a** Shake-flask fermentation of PP310 strain, which has one additional copy of the *Pp*Ilv6_T2A_*Pp*Ilv2 expression cassette integrated into the yeast chromosome. **b** Shakeflask fermentation of PP302 strain, which contains additional copies of isobutanol pathway genes *LlkivD* and *ScADH7* on an episomal plasmid. **c** Shake-flask fermentation of PP303 strain, which contains additional copies of isobutanol pathway genes *PpIlv5* and *PpIlv3* on an episomal plasmid. **d** Shake-flask fermentation of PP304 strain, which contains additional copies of isobutanol pathway genes *PpIlv6* and *PpIlv2* on an episomal plasmid. The engineered strains were pre-cultured in 5-mL aliquots in MGY (2% glycerol) minimal medium overnight and used to inoculate 50 mL fresh MGY_glu (10% glucose) in 250-mL Erlenmeyer flasks to achieve an initial optical density of 0.05 at 600 nm (OD600). The cultures were grown at 30 °C and 250 rpm in an orbital shaking incubator. Samples were taken at several time points and the supernatants were analyzed on HPLC to quantify the levels of isobutanol, glucose and other metabolites. Values are the mean of three biological replicates ± standard deviation (*n* = 3)

To improve the isobutanol production titer in PP300, we turned our attention to optimizing the expression levels of the biosynthetic pathway genes. First, we integrated an additional copy of the *Pp*Ilv6-T2A-*Pp*Ilv2 gene construct to create strain PP310 and observed an isobutanol production titer of 1.70 ± 0.07 g/L, a 92% improvement from the level observed in PP300 (at 885 ± 12 mg/L) (Fig. 8). Strain PP310 also appears to be more efficient at consuming the substrate glucose than strain PP300, with no glucose remaining in the medium after 72 h for PP310 compared to 44 ± 1 g/L (44% of the starting concentration) for PP300 (Figs. 7, 8). These results indicate that overexpression of the first enzymatic step of the l-valine pathway, acetolactate synthesis, resulted in the necessary “pull” of the cell’s metabolism towards isobutanol production. Moreover, we hypothesize that acetolactate synthesis is a potential bottleneck in isobutanol production and warrants further optimization (Fig. 3). To this end, we examined the possibility of using an episomal plasmid-based expression system. Industrial applications of *P. pastoris* as a recombinant protein production host predominantly rely on the integration of foreign expression cassette(s) at a specific site within the host’s genome. In some cases, multiple integration events can occur, which can be selected for using a higher dose of antibiotics. While chromosomal integration has the advantage over plasmid-based expression in terms of genomic stability, the latter offers a broader range of foreign gene expression levels. Indeed, having a suite of plasmids with a choice of low, medium or high copy-number has enabled scientists to fine-tune the expression levels of the individual biosynthetic enzymes that resulted in large improvement in production yields of a variety of chemicals in engineered *E. coli* and *S. cerevisiae* [29, 30].

To broaden the genetic toolbox for engineering the yeast *P. pastoris*, we created a set of episomal expression vectors by utilizing the recently discovered autonomously replicating sequence (panARS) from *Kluyveromyces lactis* (Additional file 1: Figure S4) [31, 32]. Camattari et al. demonstrated that vectors containing this 452-nt element outperformed the traditional integrative plasmids in heterologous enzyme expression, both in productivity as well as clonal homogeneity [31]. We expanded this previous work by introducing the panARS sequence into four different expression plasmids: pGAPz (for zeocin selection), pGAPhyg (for hygromycin selection), pGAPneo (for G418 selection) and pGAPHis4 (for histidine auxotrophy). All of these plasmids use the strong constitutive promoter from the glyceraldehyde-3-phosphate dehydrogenase gene (P_GAP_) to express recombinant proteins.

Using PP300 as the parental strain, we introduced additional copies of the *Pp*Ilv5_T2A_*Pp*Ilv3, *Pp*Ilv6_T2A_*Pp*Ilv2, and *Ll*kivD_T2A_*Sc*ADH7 expression cassettes by placing them on the episomal expression vector pGAPHis*Kl*ARS and transforming the individual plasmids into PP300. The resulting strains, PP302, PP303 and PP304, showed superior performance to the parental strain. In particular, PP304, which harbors the episomal plasmid containing the *Pp*Ilv6_T2A_*Pp*Ilv2 expression cassette, produced the highest titer of isobutanol at 2.22 ± 0.05 g/L (isobutanol yield of 22.2 ± 0.1 mg/g glucose, specific isobutanol production titer of 153 ± 9 mg/L/OD_600_). To the best of our knowledge, this is the highest reported isobutanol production titer and yield in a yeast system.

As was also the case for strain PP310, all strains harboring the episomal plasmids that contain additional copies of isobutanol biosynthetic pathway genes exhibited improved glucose consumption compared to strain PP300 (Figs. 7b, 8). Additionally, we observed elevated levels of the competing side-product ethanol (more than 10 g/L) in strains PP302, PP303 and PP304 when compared to the control strain KM71 (~ 3 g/L). The isobutanol pathway produces excess NADH (from glycolysis) and requires additional NADPH (from the reduction of isobutanal to isobutanol). Large accumulation of ethanol indicates that isobutanol production potentially caused redox cofactor imbalances, which ultimately forced the cells to use up the excess NADH by producing ethanol. Similar results have been observed in other microbial systems, particularly the Crabtree-positive *S. cerevisiae.* For example, Matsuda and coworkers have engineered *S. cerevisiae* to produce 1.6 g/L of isobutanol (16 mg/g glucose) by re-localizing the upper portion of valine metabolism to the cytosol and overexpressing the keto acid degradation pathway [11]. The authors observed that the strain also accumulated high levels of ethanol, at approximately 40 g/L, 25-folds the level of the desired product isobutanol. Several strategies have led to lower ethanol accumulation and improvement in isobutanol production in *S. cerevisiae*. These include: (1) downregulating the pyruvate decarboxylase enzyme [12]; (2) overexpression of a transhydrogenase or constructing a transhydrogenase-like shunt to convert excess NADH to NADPH [11]; and (3) replacing the alcohol dehydrogenase with an NADH-dependent engineered mutant [33]. These strategies could potentially improve isobutanol production in *P. pastoris.*

### Overexpression of a broad-substrate-range alcohol *O*-acyltransferase to produce acetate esters of isobutanol and other higher branched-chain alcohols

**Fig. 9:**
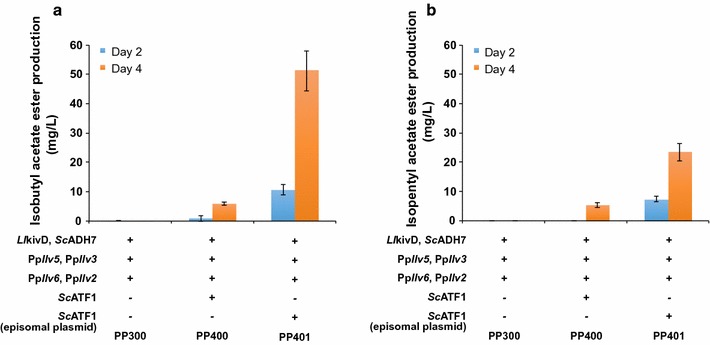
Production of isobutyl acetate (**a**) and isoamyl acetate (**b**) in engineered *P. pastoris.* Engineered strains were pre-cultured in 5-mL aliquots in MGYH minimal medium overnight and used to inoculate 5 mL fresh MGYH (with 10% glucose as the main carbon source) to achieve an initial optical density of 0.05 at 600 nm (OD600). The cultures were overlayed with 5 mL hexadecane and grown at 30 °C and 250 rpm in an orbital shaking incubator. The hexadecane layer from each sample was taken at two different time points (48 and 96 h) and the samples were analyzed on GC–MS to quantify the isobutyl acetate ester and isopentyl acetate ester content

To demonstrate the versatility of our yeast platform, we further engineered *P. pastoris* to convert isobutanol to the volatile ester isobutyl acetate. Many volatile esters exist in nature and their biosynthesis typically involves the condensation of an acyl-CoA with an alcohol. This reaction is carried out by the enzyme alcohol *O*-acyltransferases (ATFs) [34]. Along with the wax ester synthases/acyl-CoA:diacylglycerol acyltransferase (WS/DGAT), ATFs form a class of enzymes that use acyl-CoA units as the acid component for ester formation. The Baker’s yeast *S. cerevisiae*, which produces several volatile esters during beer and wine fermentation, contains four alcohol O-acyltransferase genes, *ATF1*, *ATF2*, *EHT1* and *EEB1* [35]. Previous work by Rodriguez and coworkers found that heterologous expression of *ATF1*, which has the broadest substrate scope of the four, in an engineered *E. coli* strain led to production of acetate ester of several alcohols including isobutanol and isopentanol [27]. Encouraged by these results, we constitutively expressed *S. cerevisiae ATF1* in PP300. The resulting strain (PP400) produced isobutyl acetate at a titer of 6.0 ± 0.6 mg/L, along with smaller quantities of several other acetate esters such as isoamyl acetate (Fig. 9a, b). The low titers of isobutyl acetate ester, as well as the large accumulation of isobutanol in the production culture, indicated that the conversion of isobutanol to isobutyl acetate is a potential bottleneck, indicating the need for further strain optimization. This prompted us to overexpress *ScATF1* on an episomal plasmid instead of integrating the expression cassette into the genome. The resulting strain, PP401, produced isobutyl acetate at 51.2 ± 6.8 mg/L as well as isoamyl acetate ester at 23.5 ± 3.0 mg/L (Fig. 9a, b). The episomal plasmid was stably maintained during the fermentation as confirmed by RT-PCR quantification of gene copy number (Additional file 1: Figure S6). The elevated production levels of the acetate esters correspond to the increased expression of *Sc*ATF1 and the higher gene copy number as indicated by real-time PCR (Additional file 1: Figure S5).

## Conclusions

In this work, we engineered *P. pastoris*, an industrial powerhouse in enzyme production, to produce the biofuel isobutanol and the volatile isobutyl acetate from glucose and glycerol, both of which are simple and renewable carbon sources. Our strategy exploited the yeast’s endogenous amino acid biosynthetic pathway and diverted the amino acid intermediates to the 2-keto acid degradation pathway for higher alcohol production. The engineered strain overexpressing the keto acid degradation pathway was able to produce 284 mg/L of isobutanol when supplemented with the pathway intermediate KIV. Further yield improvement was accomplished by overexpressing a portion of the amino acid l-valine biosynthetic pathway, leading to the engineered strain PP304 that is able to produce 2.22 g/L of isobutanol directly from glucose without the addition of 2-KIV to the culture medium. Finally, to demonstrate the versatility of our yeast platform, we further incorporated a broad-substrate-range alcohol *O*-acyltransferase to generate isobutyl acetate along with smaller quantities of other acetate esters. Episomal plasmid-based expression of the alcohol *O*-acyltransferase improved the production titer of isobutyl acetate to 51.2 mg/L, a ninefold improvement over the integrative plasmid-based expression. We envision that our work will pave the way for an economic route to this important class of compounds and establish *P. pastoris* as a versatile production platform for fuels and chemicals.

## Additional file

**Additional file 1.** Supplementary information for metabolic engineering of the methylotrophic yeast pichia pastoris for production of isobutanol and isobutyl acetate.
