# Supplementary material for: Metabolic engineering of Pichia pastoris for production of isobutanol and isobutyl acetate
Source: Biotechnol Biofuels. 2018 Jan 8;11:1. doi: 10.1186/s13068-017-1003-x (PMC5757298; doi:10.1186/s13068-017-1003-x)
Supplement: Supplementary file 1 — Additional file 1. Supplementary information for metabolic engineering of the methylotrophic yeast pichia pastoris for production of isobutanol and isobutyl acetate. [file 13068_2017_1003_MOESM1_ESM.docx]

**Supplementary Information for Metabolic Engineering of the Methylotrophic Yeast *Pichia pastoris* for Production of Isobutanol and Isobutyl Acetate**

Wiparat Siripong^1^, Philipp Wolf^2^, Theodora Puspowangi Kusumoputri^3^, Joe James Downes^4^, Kanokarn Kocharin^1^, Sutipa Tanapongpipat^1^, and Weerawat Runguphan^1^

^1^National Center for Genetic Engineering and Biotechnology, 113 Thailand Science Park, Paholyothin Road, Klong 1, Klong Luang, Pathumthani 12120, Thailand

^2^ Leipzig University, Brüderstraße 34, 04103 Leipzig, Germany

^3^ Atma Jaya University, Jl. Jend. Sudirman No.51, RT.5/RW.4, Karet Semanggi, Setia Budi, Kota Jakarta Selatan, Daerah Khusus Ibukota Jakarta 12930, Indonesia

^4^University of Kent, Canterbury, Kent, UK

**Table 1.** Strains used in this study.

| Strain name | Genotype | Description | Reference |
| --- | --- | --- | --- |
| KM71 | arg4; his4; aox1::ARG4 | none | Invitrogen |
| PP010 | arg4; his4; aox1::ARG4; P_GAP_-LlkivD | KM71 with overexpression of *LlkivD* | This study |
| PP011 | arg4; his4; aox1::ARG4; P_GAP_-LlkivD; P_GAP_-ScADH6 | KM71 with overexpression of *LlkivD* and *ScADH6* | This study |
| PP012 | arg4; his4; aox1::ARG4; P_GAP_-LlkivD; P_GAP_-ScADH7 | KM71 with overexpression of *LlkivD* and *ScADH7* | This study |
| PP020 | arg4; his4; aox1::ARG4; P_GAP_-ScARO10 | KM71 with overexpression of *ScARO10* | This study |
| PP021 | arg4; his4; aox1::ARG4; P_GAP_-ScARO10; P_GAP_-ScADH6 | KM71 with overexpression of *ScARO10* and *ScADH6* | This study |
| PP022 | arg4; his4; aox1::ARG4; P_GAP_-ScARO10; P_GAP_-ScADH7 | KM71 with overexpression of *ScARO10* and *ScADH7* | This study |
| PP030 | arg4; his4; aox1::ARG4; P_GAP_-ScTHI3 | KM71 with overexpression of *ScTHI3* | This study |
| PP031 | arg4; his4; aox1::ARG4; P_GAP_-ScTHI3; P_GAP_-ScADH6 | KM71 with overexpression of *ScTHI3* and *ScADH6* | This study |
| PP032 | arg4; his4; aox1::ARG4; P_GAP_-ScTHI3; P_GAP_-ScADH7 | KM71 with overexpression of *ScTHI3* and *ScADH7* | This study |
| PP100 | arg4; his4; aox1::ARG4; P_GAP_-LlkivD-T2A-ScADH7 | KM71 with overexpression of *LlkivD* and *ScADH7* | This study |
| PP110 | arg4; his4; aox1::ARG4; P_GAP_-LlkivD-T2A-ScADH7; P_GAP_-ScIlv2-T2A-ScIlv5-ScIlv3 | KM71 with overexpression of LlkivD, ScADH7, ScIlv2, ScIlv5 and ScIlv3 | This study |
| PP200 | arg4; his4; aox1::ARG4; P_GAP_-LlkivD-T2A-ScADH7; P_GAP_-PpIlv5-T2A-PpIlv3 | KM71 with overexpression of *LlkivD*, *ScADH7, PpIlv5* and *PpIlv3* | This study |
| PP300 | arg4; his4; aox1::ARG4; P_GAP_-LlkivD-T2A-ScADH7; P_GAP_-PpIlv5-T2A-PpIlv3; P_GAP_-PpIlv6-T2A-PpIlv2 | KM71 with overexpression of *LlkivD*, *ScADH7, PpIlv5, PpIlv3, PpIlv6* (codon optimized) and *PpIlv2* (codon optimized) | This study |
| PP310 | arg4; his4; aox1::ARG4; P_GAP_-LlkivD-T2A-ScADH7; P_GAP_-PpIlv5-T2A-PpIlv3; P_GAP_-PpIlv6-T2A-PpIlv2; pGAPHis4-KlARS-PpIlv6-T2A-PpIlv2 | KM71 with overexpression of *LlkivD*, *ScADH7, PpIlv5, PpIlv3, PpIlv6* (codon optimized) and *PpIlv2* (codon optimized) and further overexpression of *PpIlv6* and *PpIlv2* by integration of another copy of the gene cassette | This study |
| PP302 | arg4; his4; aox1::ARG4; P_GAP_-LlkivD-T2A-ScADH7; P_GAP_-PpIlv5-T2A-PpIlv3; P_GAP_-PpIlv6-T2A-PpIlv2 harboring pGAPHis4-KlARS-LlkivD-T2A-ScADH7 | KM71 with overexpression of *LlkivD*, *ScADH7, PpIlv5, PpIlv3, PpIlv6* (codon optimized) and *PpIlv2* (codon optimized) and further episomal-plasmid based expression of *LlkivD* and *ScADH7* | This study |
| PP303 | arg4; his4; aox1::ARG4; P_GAP_-LlkivD-T2A-ScADH7; P_GAP_-PpIlv5-T2A-PpIlv3; P_GAP_-PpIlv6-T2A-PpIlv2 harboring pGAPHis4-KlARS-PpIlv5-T2A-PpIlv3 | KM71 with overexpression of *LlkivD*, *ScADH7, PpIlv5, PpIlv3, PpIlv6* (codon optimized) and *PpIlv2* (codon optimized) and further episomal-plasmid based expression of *PpIlv5* and *PpIlv3* | This study |
| PP304 | arg4; his4; aox1::ARG4; P_GAP_-LlkivD-T2A-ScADH7; P_GAP_-PpIlv5-T2A-PpIlv3; P_GAP_-PpIlv6-T2A-PpIlv2 harboring pGAPHis4-KlARS-PpIlv6-T2A-PpIlv2 | KM71 with overexpression of *LlkivD*, *ScADH7, PpIlv5, PpIlv3, PpIlv6* (codon optimized) and *PpIlv2* (codon optimized) and further episomal-plasmid based expression of *PpIlv6* and *PpIlv2* | This study |
| PP400 | arg4; his4; aox1::ARG4; P_GAP_-LlkivD-T2A-ScADH7; P_GAP_-PpIlv5-T2A-PpIlv3; P_GAP_-PpIlv6-T2A-PpIlv2; P_GAP_-ScATF1 | KM71 with overexpression of *LlkivD*, *ScADH7, PpIlv5, PpIlv3, PpIlv6* (codon optimized)*, PpIlv2* (codon optimized) and *ScATF1* | This study |
| PP401 | arg4; his4; aox1::ARG4; P_GAP_-LlkivD-T2A-ScADH7; P_GAP_-PpIlv5-T2A-PpIlv3; P_GAP_-PpIlv6-T2A-PpIlv2; harboring pGAPHis4-KlARS-ScATF1 | KM71 with overexpression of *LlkivD*, *ScADH7, PpIlv5, PpIlv3, PpIlv6* (codon optimized)*, PpIlv2* (codon optimized) and episomal-plasmid based expression of *ScATF1* | This study |

**Primers used in this study**

The Kozak sequence is underlined.

Plasmid pGAPNeo:

S1. Forward:

5’ ACAAGGTGAGGAACTAAACCATGGGTAAGGAAAAGACTC 3’

S2. Reverse:

5’ GTGGGCCGCCGTCGGACGTGTTAGAAAAACTCATCGAGC 3’

S3. Forward:

5’ CACGTCCGACGGCGGCCC 3’

S4. Reverse:

5’ GGTTTAGTTCCTCACCTTG 3’

Plasmid pGAPHyg:

S5. Forward:

5’ ACAAGGTGAGGAACTAAACCATGGGTAAAAAGCCTGAAC 3’

S6. Reverse:

5’ GTGGGCCGCCGTCGGACGTGTTATTCCTTTGCCCTCGG 3’

Plasmid pGAPHis4:

S7. Forward:

5’ TGAAAAATAACAGTTATTATTCGAGATCTTTTTTGTAGAAATGTCTTGG 3’

S8. Reverse:

5’ CCGCATTAAAGCTTATCGATGGATCCGCACAAACGAAG 3’

S9. Forward:

5’ ATCGATAAGCTTTAATGCG 3’

S10. Reverse:

5’ CGAATAATAACTGTTATTTTTCAGTGTTCCC 3’

Plasmid pGAPzLlkivD:

S11. Forward:

5’ ATATATGGTACCGAAACGATGTACACTGTCGGAGAT 3’

S12. Reverse:

5’ ATATATGCGGCCGCTTAGGATTTGTTCTGTTC 3’

Plasmid pGAPhygScADH6:

S13. Forward:

5’ ATATATGGTACCGAAACGATGTCTTATCCTGAGAAATTTG 3’

S14. Reverse:

5’ ATATATGCGGCCGCCTAGTCTGAAAATTCTTTGTC 3’

Plasmid pGAPhygScADH7:

S15. Forward:

5’ ATATATGGTACCGAAACGATGCTTTACCCAGAAAAATTTC 3’

S16. Reverse:

5’ ATATATGCGGCCGCCTATTTATGGAATTTCTTATCATA 3’

Plasmid pGAPzLlkivD-T2A-ScADH7:

S17. Forward:

5’ ACAAACAAACCGCGGAAAACAATGTACACTGTCGGA 3’

S18. Reverse:

5’ CTCAACATCTCCACAAGTCAACAAAGAACCCCTTCCTTCTGCTCTGGATTTGTTC TGTTCTGCGAA 3’

S19. Forward:

5’ GGTTCTTTGTTGACTTGTGGAGATGTTGAGGAGAATCCAGGACCAATGCTTTAC CCAGAAAAATTT 3’

S20. Reverse:

5’ GACGGTATCGATAAGCTTGATATCGAATTCCTATTTATGGAATTTCTTATCATA 3’

S21. Forward:

5’ ATACGACTCACTATAGGGAGACCGGCAGATCCGCGGGAGTTTATCATT 3’

S22. Reverse:

5’ AGTGTACATTGTTTTCCGCGGTTTGTTTGTTTA 3’

S23. Forward:

5’ TATAGGTACCGAAACGATGTACACTGTCGGAG 3’

S24. Reverse:

5’ ATATGCGGCCGCCTATTTATGGAATTTCTTATCATA 3’

pGAPNeoScIlv2-T2A1-ScIlv5-T2A2-ScIlv3:

S25. Forward:

5’ CTTGCTCATTAGAAAGAAAGCATAGCAATCTAATCTAAGTTTTCTAGAACTAGTG GATCCAAAACAATGATCAGACAATCTACGCTA 3’

S26. Reverse:

5’ TGGTCCTGGATTCTCCTCAACATCTCCACAAGTCAACAAAGAACCCCTTCCTTC TGCTCTGTGCTTACCGCCTGTACG 3’

S27. Forward:

5’ AGAGCAGAAGGAAGGGGTTCTTTGTTGACTTGTGGAGATGTTGAGGAGAATCC AGGACCATTGAGAACTCAAGCCGCC 3’

S28. Reverse:

5’ AGGACCAGGGTTTTCTTCTACGTCACCGCATGTTAGTAGACTTCCTCTACCCTC AGCTCTTTGGTTTTCTGGTCTCAACTT 3’

S29. Forward:

5’ AGAGCTGAGGGTAGAGGAAGTCTACTAACATGCGGTGACGTAGAAGAAAACCC TGGTCCTGGCTTGTTAACGAAAGTTGCT 3’

S30. Reverse:

5’ GTGACATAACTAATTACATGACTCGAGGTCGACGGTATCGATAAGCTTGATATC GAATTCTCAAGCATCTAAAACACAACC 3’

S31. Forward:

5’ ATATATCTCGAGGAAACGATGATCAGACAATCTACGCTA 3’

S32. Reverse:

5’ ATATATGCGGCCGCTCAAGCATCTAAAACACAACC 3’

Plasmid pGAPHygPpIlv5-T2A1-PpIlv3:

S33. Forward:

5’ GCAATCTAATCTAAGTTTTCTAGAACTAGTGGATCCATGTCCGTAAGAAATGCC AC 3’

S34. Reverse:

5’ GACATCACCACATGTCAACAAACTACCTCTACCTTCGGCTCTGTTGTTTTCTGG ACGTAG 3’

S35. Forward:

5’ AGTTTGTTGACATGTGGTGATGTCGAAGAAAATCCAGGTCCAAATATTGCGACT CGTGCC 3’

S36. Reverse:

5’ GAGGTCGACGGTATCGATAAGCTTGATATCGAATTCTTAGTAGTAGTCAGCGTC 3’

S37. Forward:

5’ ATATATCTCGAGGAAACGATGTCCGTAAGAAATGCC 3’

S38. Reverse:

5’ ATATATGCGGCCGCTTAGTAGTAGTCAGCGTCC 3’

Plasmid pGAPNeoPpIlv2a-T2A1-PpIlv2b:

S39. Forward:

5’ ATATATGAATTCGAAACGATGTCCGCTGGAAGATTA 3’

S40. Reverse:

5’ ATATATGCGGCCGCTTAGTGCTTACCATTGGTAC 3’

Plasmid pGAPHis4-ScATF1:

S41. Forward:

5’ ATATGCGGCCGCGAAACGATGAATGAAATCGATGAGAA 3’

S42. Reverse:

5’ ATATGGGCCCCTAAGGGCCTAAAAGGAG 3’

Plasmid pGAPHis4-*Kl*ARS:

S43. Forward:

5’ ATATATGACGTCTCAACATCTTTGGATAATATC 3’

S44. Reverse:

5’ ATATATGACGTCTAGTGCTGATTATGATTTG 3’

Plasmid pGAPZ-*Kl*ARS:

S45. Forward:

5’ ATATATATGCATTCAACATCTTTGGATAATATC 3’

S46. Reverse:

5’ ATATATAGATCTTAGTGCTGATTATGATTTG 3’

Plasmid pGAPHis4-*Kl*ARS-LlkivD-T2A-ScADH7:

S47. Forward:

5’ ATATGCGGCCGCGAAACGATGTACACTGTCGGA 3’

S48. Reverse:

5’ ATATGGGCCCCTATTTATGGAATTTCTTATCATAATC 3’

Plasmid pGAPHis4-*Kl*ARS-PpIlv5-T2A1-PpIlv3:

S49. Forward:

5’ ATATGCGGCCGCGAAACGATGTCCGTAAGAAATGCC 3’

S50. Reverse:

5’ ATATGGGCCCTTAGTAGTAGTCAGCGTCC 3’

Real-time PCR primers

PpIlv5-RT Forward:

5’ TGGTCAGAATTGCTTCTCAAGCCT 3’

PpIlv5-RT Reverse:

5’ CGTGGACAACTTCTTCGACACC 3’

PpIlv3-RT Forward:

5’ CCGGTCAGATCACCGAGGAG 3’

PpIlv3-RT Reverse:

5’ TCACCTCAGCACAGGATGCC 3’

PpIlv6_native RT Forward:

5’ CTTTGCCCACGTTGGAGACC 3’

PpIlv6_native RT Reverse:

5’ CGTGCTCGGTGGAAGGATCT 3’

PpIlv2_native RT Forward:

5’ ACATTGACGGTGACGCATCC 3’

PpIlv2_native RT Reverse:

5’ GCCATTGGGTGACCATTCCC 3’

PpIlv6-T2A-PpIlv2_codon opt RT Forward:

5’ AAGGTGCTGGTCACATGGCT 3’

PpIlv6-T2A-PpIlv2_codon opt RT Reverse:

5’ GGAACACCATCAGCCAAAGCA 3’

LlkivD-T2A-ScADH7 RT Forward:

5’ TCGCAGAACAGAACAAATCC 3’

LlkivD-T2A-ScADH7 RT Reverse:

5’ AAATACCGATGCCCTGAAAT 3’

PpACT1-RT Forward:

5’ ACAGTGTTCCCATCGGTCGT 3’

PpACT1-RT Reverse:

5’ GGATTGAGCCTCGTCACCGA 3’

**Plasmid construction**

Plasmid pGAPNeo:

The Neo (NPTII) selection marker was amplified from pUG6 using primers S1 and S2 (*22*). The vector backbone containing the pGAP expression cassette was amplified from pGAPZ_A (Invitrogen) using primers S3 and S4. The two fragments were assembled together by using New England Biolab’s NEBuilder HiFi DNA Assembly Kit to yield pGAPNeo.

Plasmid pGAPHyg:

The hygromycin selection marker was amplified from pUG75 using primers S5 and S6 (*22*). The vector backbone containing the pGAP expression cassette was amplified from pGAPZ_A (Invitrogen) using primers S3 and S4. The two fragments were assembled together by using New England Biolab’s NEBuilder HiFi DNA Assembly Kit to yield pGAPhyg.

Plasmid pGAPHis4:

The P_GAP_ promoter-MCS-AOX1 terminator cassette was amplified from pGAPz plasmid using primers S7 and S8. The vector backbone containing the HIS4 selection marker was amplified from pPIC3.5K (Thermo Fisher Scientific) using primers S9 and S10. The two fragments were assembled together by using New England Biolab’s NEBuilder HiFi DNA Assembly Kit.

Plasmid pGAPZ-LlkivD:

The keto acid decarboxylase gene *LlkivD* codon-optimized for *P. pastoris* expression was synthesized by GenScript and was provided as a pUC57 plasmid. The gene was amplified from the pUC57-LlkivD using primers S11 and S12. The Kozak sequence GAAACG was added 5′ of the start codon to enhance expression. The amplicon was ligated to the KpnI/NotI site of pGAPZ_A.

Plasmid pGAPHyg-ScADH6:

The alcohol dehydrogenase gene *ScADH6* was amplified from *S. cerevisiae* genomic DNA using primers S13 and S14. The Kozak sequence GAAACG was added 5′ of the start codon to enhance expression. The amplicon was ligated to the KpnI/NotI site of pGAPhyg.

Plasmid pGAPHyg-ScADH7:

The alcohol dehydrogenase gene *ScADH7* was amplified from *S. cerevisiae* genomic DNA using primers S15 and S16. The Kozak sequence GAAACG was added 5′ of the start codon to enhance expression. The amplicon was ligated to the KpnI/NotI site of pGAPhyg.

Plasmid pGAPZ-LlkivD-T2A-ScADH7:

The keto acid decarboxylase gene *LlkivD* and the alcohol dehydrogenase gene *ScADH7* were amplified from pUC57-LlkivD and *S. cerevisiae* genomic DNA using primers S17 and S18, S19 and S20, respectively. Each primer contains the DNA sequence encoding the T2A peptide. The P_TDH3_ promoter from *S. cerevisiae* was amplified from *S. cerevisiae* genomic DNA using primers S21 and S22. Gibson assembly using the three fragments and the SacII/EcoRI digested pUG72 vector yielded pUG72-pTDH3-LlkivD-T2A-ScADH7. The LlkivD-T2A-ScADH7 gene fusion was then amplified from pUG72-pTDH3-LlkivD-T2A-ScADH7 using primers S23 and S24, and the amplicon was ligated to the KpnI/NotI digested pGAPZ_A.

Plasmid pGAPNeo-ScIlv2-T2A1-ScIlv5-T2A2-ScIlv3:

The *S. cerevisiae* valine biosynthetic pathway genes *ScIlv2, ScIlv5* and *ScIlv3* were amplified from *S. cerevisiae* genomic DNA using primers S25 and S26, S27 and S28, S29 and S30, respectively. Each primer contains the DNA sequence encoding the T2A peptide. Homologous recombination in *S. cerevisiae* BY4742 using the three fragments and the BamHI/EcoRI digested pRS416Tef1 (*23*) yielded pRS416Tef1-ScIlv2-T2A1-ScIlv5-T2A2-ScIlv3. Yeast transformants were checked with colony PCR to verify proper assembly. Plasmids were harvested from selected colonies and transformed into *E. coli* for maintenance and propagation. The ScIlv2-T2A1-ScIlv5-T2A2-ScIlv3 gene fusion was then amplified from pRS416Tef1-ScIlv2-T2A1-ScIlv5-T2A2-ScIlv3 using primers S31 and S32, and the amplicon was ligated to the XhoI/NotI digested pGAPNeo.

Plasmid pGAPHyg-PpIlv5-T2A1-PpIlv3:

The *P. pastoris* valine biosynthetic pathway genes *PpIlv5* and *PpIlv3* were amplified from *P. pastoris* genome DNA using primers S33 and S34, S35 and S36, respectively. Each primer contains the DNA sequence encoding the T2A peptide. Homologous recombination in *S. cerevisiae* BY4742 using the two fragments and the BamHI/EcoRI digested pRS416Tef1 (*23*) yielded pRS416Tef1-PpIlv5-T2A1-PpIlv3. Yeast transformants were checked with colony PCR to verify proper assembly. Plasmids were harvested from selected colonies and transformed into *E. coli* for maintenance and propagation. The PpIlv5-T2A1-PpIlv3 gene fusion was then amplified from pRS416Tef1-PpIlv5-T2A1-PpIlv3 using primers S37 and S38, and the amplicon was ligated to the XhoI/NotI digested pGAPHyg.

Plasmid pGAPNeo-PpIlv6-T2A1-PpIlv2:

The *P. pastoris* valine biosynthetic pathway genes *PpIlv6* and *PpIlv2* linked by the DNA sequence for T2A peptide were synthesized by GenScript and was provided as a pCCI vector. The PpIlv6-T2A-PpIlv2 construct was amplified from pCCI-PpIlv6-T2A1-PpIlv2 using primers S39 and S40, and the amplicon was ligated to the EcoRI/NotI digested pGAPNeo.

Plasmid pGAPHis4-ScATF1:

The alcohol O-acyltransferase gene *ScATF1* was amplified from *S. cerevisiae* genomic DNA using primers S41 and S42. The Kozak sequence GAAACG was added 5′ of the start codon to enhance expression. The amplicon was ligated to the Not/ApaI site of pGAPHis4.

Plasmid pGAPHis4-*Kl*ARS:

The autonomously replicating sequence (ARS) from *Kluyveromyces lactis* was amplified from *K. lactis* TBRC 890 genomic DNA using primers S43 and S44, and the amplicon was ligated to the ApaI site of pGAPHis4.

Plasmid pGAPZ-*Kl*ARS:

The autonomously replicating sequence (ARS) from *K. lactis* was amplified from *K. lactis* TBRC 890 genomic DNA using primers S45 and S46, and the amplicon was ligated to the NsiI/BglII site of pGAPZ_A.

Plasmid pGAPHyg-*Kl*ARS:

The autonomously replicating sequence (ARS) from *K. lactis* was amplified from *K. lactis* TBRC 890 genomic DNA using primers S45 and S46, and the amplicon was ligated to the NsiI/BglII site of pGAPHyg.

Plasmid pGAPNeo-*Kl*ARS:

The autonomously replicating sequence (ARS) from *K. lactis* was amplified from *K. lactis* TBRC 890 genomic DNA using primers S45 and S46, and the amplicon was ligated to the NsiI/BglII site of pGAPNeo.

Plasmid pGAPHis4-*Kl*ARS-ScATF1:

The alcohol O-acyltransferase gene *ScATF1* was amplified from *S. cerevisiae* genomic DNA using primers S41 and S42. The Kozak sequence GAAACG was added 5′ of the start codon to enhance expression. The amplicon was ligated to the Not/ApaI site of pGAPHis4-*Kl*ARS.

Plasmid pGAPHis4-*Kl*ARS-LlkivD-T2A-ScADH7:

The LlkivD-T2A-ScADH7 gene fusion was then amplified from pUG72-pTDH3-LlkivD-T2A-ScADH7 using primers S47 and S48, and the amplicon was ligated to the NotI/ApaI digested pGAPHis4-*Kl*ARS.

Plasmid pGAPHis4-*Kl*ARS-PpIlv5-T2A1-PpIlv3:

The PpIlv5-T2A1-PpIlv3 gene fusion was then amplified from pRS416Tef1-PpIlv5-T2A1-PpIlv3 using primers S49 and S50, and the amplicon was ligated to the NotI/ApaI digested pGAPHis4-*Kl*ARS.

Plasmid pGAPHis4-*Kl*ARS-PpIlv6-T2A1-PpIlv2:

The PpIlv6-T2A-PpIlv2 construct was amplified from pCCI-PpIlv6-T2A1-PpIlv2 using primers S39 and S40, and the amplicon was ligated to the EcoRI/NotI digested pGAPHis4-*Kl*ARS.

**Figure S1. Production of isobutanol in engineered *P. pastoris****.* Engineered strains were pre-cultured in 5-mL aliquots in MGYH minimal medium overnight and used to inoculate 5 mL fresh MGYH (with glycerol as the main carbon source) to achieve an initial optical density of 0.05 at 600 nm (OD_600_). The medium also contained 2 g/L 2-ketoisovalerate. The cultures were grown at 30 ºC and 250 rpm in an orbital shaking incubator. Samples were taken at four different time points and the supernatants were analyzed on HPLC to quantify the isobutanol content. The strain PP100 (KM71 harboring pGAPZLlkivD pGAPHygScADH7) was used as a control strain.

**Figure S2. Effects of carbon source on expression levels of isobutanol biosynthetic pathway genes.** The engineered strain PP300 was pre-cultured in 5-mL aliquots in MGYH (2% glycerol) minimal medium overnight and used to inoculate either 10 mL fresh MGYH (2% glycerol) or 10 mL fresh MGYH_glu (2% glucose) to achieve an initial optical density of 0.05 at 600 nm (OD_600_). The cultures were grown at 30 ºC and 250 rpm in an orbital shaking incubator. Samples were taken at the 48 hours time point for real time RT-PCR analysis.

**Figure S3. Effects of glucose concentration on gene expression.** The engineered strain PP300 was pre-cultured in 5-mL aliquots in MGYH (2% glycerol) minimal medium overnight and used to inoculate either 10 mL fresh MGYH_glu (2% glucose) or 10 mL fresh MGYH_glu (10% glucose) to achieve an initial optical density of 0.05 at 600 nm (OD_600_). The cultures were grown at 30 ºC and 250 rpm in an orbital shaking incubator. Samples were taken at the 48 hours time point for real time RT-PCR analysis.


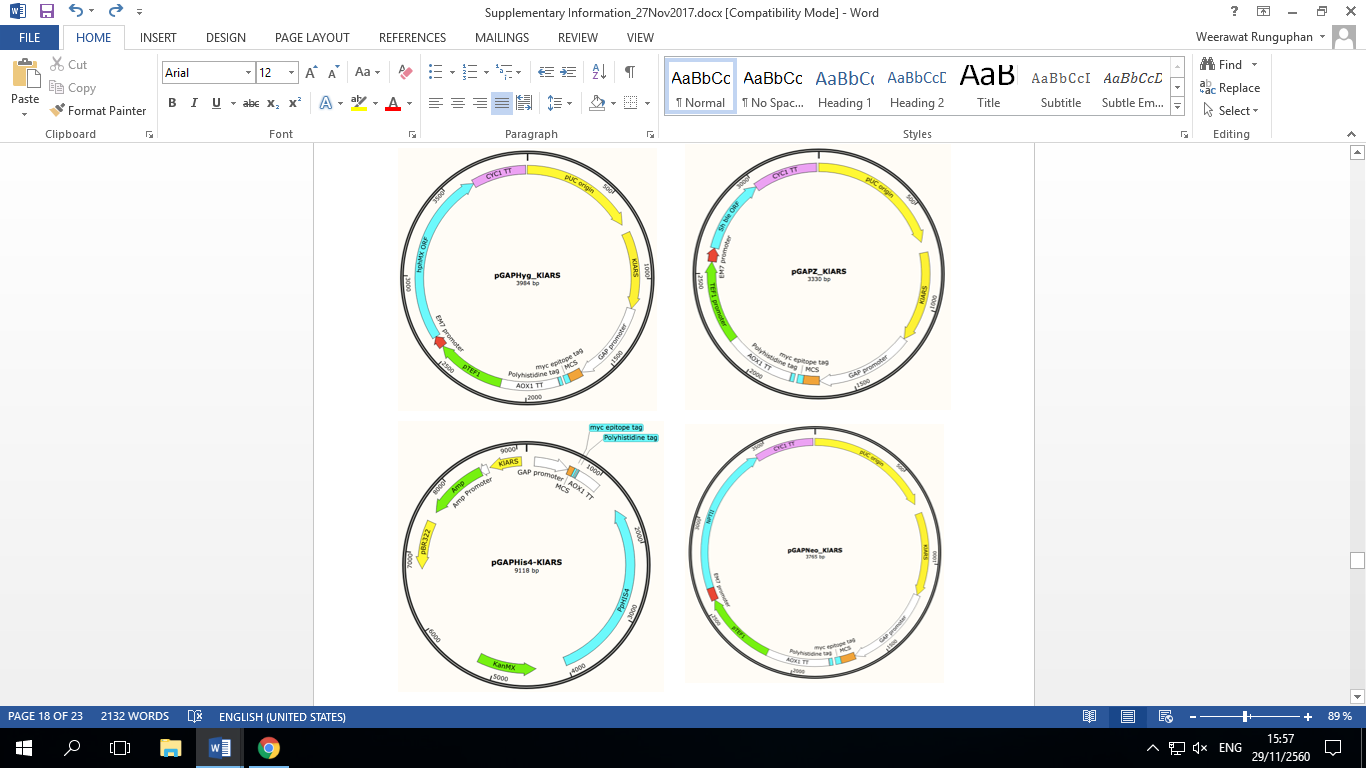


**Figure S4. Plasmids for episomal-based expression in *Pichia pastoris.***

**Figure S5. Real-time PCR analysis of *Sc*ATF1 expression (C) and gene copy number (D) in engineered *P. pastoris****.* Engineered strains were pre-cultured in 5-mL aliquots in MGYH minimal medium overnight and used to inoculate 10 mL fresh MGYH (with 10% glucose as the main carbon source) to achieve an initial optical density of 0.05 at 600 nm (OD_600_). The cultures were overlayed with 10 mL hexadecane and grown at 30 ºC and 250 rpm in an orbital shaking incubator. The cell cultures (aqueous layer) were collected after 48 hours for gene expression and gene copy number analysis.

**Figure S6. Verification of the stability of the episomal plasmid pGAPHis4-KlARS-ScATF1. (A) Plasmid isolation and restriction digestion from engineered *P. pastoris* Strain PP401. (B) Real-time PCR analysis of gene copy number in engineered *P. pastoris* Strain PP401***.* The engineered strain were pre-cultured in 5-mL aliquots in MGYH minimal medium overnight and used to inoculate 50 mL fresh MGYH (with 10% glucose as the main carbon source) to achieve an initial optical density of 0.05 at 600 nm (OD_600_). The cultures were grown at 30 ºC and 250 rpm in an orbital shaking incubator. The cell cultures were collected after 1, 2, 3 and 4 days for plasmid verification and gene copy number analysis. Real-time PCR was performed in triplicate, and normalization of the data was achieved using actin as a reference (i.e. *Pp*ACT1 gene copy number = 1). For plasmid analysis, the episomal plasmid was extracted from the yeast cultures at different time point and transformed into *E. coli* DH5alpha. Two colonies from each transformant were randomly selected for plasmid extraction. The isolated plasmids were then digested with the restriction enzyme PvuI (left panel) or HindIII (right panel).

**Figure S7. Engineered isobutanol production in *Pichia pastoris*. Biomass, isobutanol titer and glycerol formation are shown. (A)** KM71 (**B**) PP100 (**C**) PP200 (**D**) PP300 **(E)** PP300 in MGY_glu (2% glucose) media (**F**) PP310 (**G)** PP302 (**H**) PP303 (**I)** PP304. The engineered strains were pre-cultured in 5-mL aliquots in MGY (2% glycerol) minimal medium overnight and used to inoculate 50 mL fresh MGY_glu (10% glucose or 2% glucose) in 250-mL Erlenmeyer flasks to achieve an initial optical density of 0.05 at 600 nm (OD_600_). The cultures were grown at 30 ºC and 250 rpm in an orbital shaking incubator. Samples were taken at several time points and the supernatants were analyzed on HPLC to quantify the levels of isobutanol, glucose and other metabolites. Values are the mean of three biological replicates ± standard deviation (n = 3).

**Figure S8.** **Effects of isobutanol concentration on *Pichia pastoris* growth.** *P. pastoris* KM71 was pre-cultured in 5-mL YPD medium overnight and used to inoculate 10 mL fresh YPD with various concentrations of isobutanol (from 0 – 10 g/L) to achieve an initial optical density of 0.05 at 600 nm (OD_600_). The cultures were grown at 30 ºC and 250 rpm in an orbital shaking incubator. The cell cultures were collected after 24, 48, 72 and 96 hours for OD_600_ measurement.
